# Relationship between PD-L1 expression and ^18^F-FDG uptake in gastric cancer

**DOI:** 10.18632/aging.102567

**Published:** 2019-12-17

**Authors:** Ruohua Chen, Yumei Chen, Gang Huang, Jianjun Liu

**Affiliations:** 1 Department of Nuclear Medicine, Ren Ji Hospital, School of Medicine, Shanghai Jiao Tong University, Shanghai, China; 2 Shanghai University of Medicine and Health Sciences, Shanghai, China

**Keywords:** SUVmax, PD-L1, gastric cancer, PET/CT

## Abstract

Purpose: Immunotherapy has been successfully utilized for treatment of gastric cancer, so the identification of clinicopathologic features that are predictive of response to this therapy is crucial. ^18^F-FDG PET/CT can provide information on the molecular phenotype of many malignant tumors. The correlation between ^18^F-FDG accumulation and PD-L1/PD-L1-TILs status in gastric cancer patients has not been investigated. The aim of the current study is to assess whether ^18^F-FDG accumulation is associated with PD-L1/PD-L1-TILs status, and whether ^18^F-FDG PET/CT may be useful for predicting PD-L1/PD-L1-TILs expression of gastric cancer.

Results: Tumors with positive PD-L1 expression had higher SUVmax than in tumors with negative PD-L1 expression (15.0 ± 8.0 vs. 7.2 ± 4.2, respectively; P = 0.004). Tumors with positive PD-L1-TILs expression also had higher SUVmax than in tumors with negative PD-L1-TILs expression (10.3 ± 6.5 vs. 6.6 ± 3.7, respectively; P = 0.034). Multivariate analysis suggested that SUVmax remained significantly correlated with the status of PD-L1 (P = 0.043) and PD-L1-TILs (P = 0.016). PD-L1 expression was predicted with an accuracy of 67.2% when a SUVmax value of 8.55 was used as a cutoff point for analysis. Similarly, PD-L1-TILs expression was predicted with an accuracy of 64.2%, when a SUVmax value of 7.9 was used as the threshold for analysis.

Conclusion: Higher ^18^F-FDG accumulation in gastric cancers is correlated with positive PD-L1/PD-L1-TILs expression. ^18^F-FDG PET/CT may be used to predict the status of PD-L1/PD-L1-TILs and thus aid in optimal treatment decision.

Methods: A retrospective analysis was conducted on 64 patients with gastric cancer who underwent ^18^F-FDG PET/CT. SUVmax was calculated from the ^18^F-FDG accumulation of the primary tumor. The relationship between SUVmax and PD-L1/PD-L1-TILs status was analyzed.

## INTRODUCTION

Gastric cancer is the fifth most frequent malignant tumors worldwide [1]. Surgery is still the major treatment for gastric cancer. However, gastric cancer patients are often diagnosed with inoperable or metastatic disease, and treatment outcomes for such patients remain poor. Therefore, it is essential to identify and develop effective therapeutic regimens for these patients. Despite the use of Trastuzumab for patients with positive HER2 expression and targeting VEGFR2 leading to improved survival [2–4], there is still a considerable number of patients who are unresponsive to treatment. Immunotherapy has become interesting in many malignant tumors, and the PD-1/PD-L1 pathway is the main mechanism underlying immunotherapy [5, 6]. Immunotherapy with anti-PD-L1 blocker is seen as an effective therapeutic approach for many malignant tumors. Recently, pembrolizumab was approved to use in gastric cancer patients with metastatic or recurrent locally lesions and high PD-L1 expression in the USA [7]. Many studies have suggested that gastric cancer patients with high PD-L1 expression show an elevated overall response rate than those lacking PD-L1 expression [8]. Teng MW et al. showed that TIL positive/PD-L1 positive pattern and TIL positive/PD-L1 negative pattern can be regarded as "Hot tumor" which can expect therapeutic effect from PD-1 targeted therapy combined with or without another chemotherapy [9]. Recent Phase 3 clinical trials (KEYNOTE-061) concluded the case with PD-L1 combined positive score (CPS) >10 had clinically significant results by the first line PD-L1 targeted therapy in a patient with unresectable advanced gastric cancer or recurrent gastric cancer [10]. It is therefore meaningful to identify useful clinicopathologic feature in gastric cancer patients to predict PD-L1 expression. However, so far there are no validated clinicopathologic characteristics to select *a priori* patients who may benefit from immunotherapy in gastric cancer.

^18^F-FDG PET/CT is a noninvasive method to detect malignant tumors [11–13]. Our previous studies suggested that ^18^F-FDG PET/CT could be useful for predicting molecular phenotype in several malignant tumors, including LDHA in lung cancer and FBP1 expression in hepatocellular carcinoma [14, 15]. However, the relationship between ^18^F-FDG accumulation and PD-L1 status and the underlying molecular mechanisms are still unclear in gastric cancer patients.

In current study we assessed whether the PD-L1 status of tumor cells (PD-L1) or PD-L1 status of tumor infiltrating lymphocytes (PD-L1-TILs) is correlated with ^18^F-FDG accumulation. We also assessed whether ^18^F-FDG PET/CT has the potential to predict PD-L1/PD-L1-TILs status in gastric cancer. So far, our study is the first to deliver data of ^18^F-FDG PET/CT for predicting PD-L1/PD-L1-TILs expression, as well as to demonstrate that ^18^F-FDG PET/CT has a great effect on determining optimal treatment methods by predicting response to immunotherapy in gastric cancer patients.

## RESULTS

### Study population

Patients’ clinicopathologic features are shown in Table 1. Among the 64 cases, 50 were treated with total or subtotal gastrectomy with lymphadenectomy, and 14 were treated with chemotherapy. 12 patients had well/ moderately differentiated adenocarcinoma, 39 patients had poorly differentiated adenocarcinoma, 9 patients had signet-ring cell carcinoma, the other 4 patients were confirmed to have adenocarcinoma, but the differentiation grade was undetermined. The SUVmax of gastric cancer ranged from 1.8 to 27.7, with an average of 8.0. Positive PD-L1 expression was found in 10.9% (7/64) of primary tumors, and positive PD-L1-TILs expression was found in 39.1% (25/64) of tumor infiltrating lymphocytes.

**Table 1:** Patients and tumor characteristics (n=64).

**Characteristics**	**No. of patients**
**Sex**	
Male	44
Female	20
**Age (y)**	
Mean ± SD	60.9±13.2
Range	26-84
**Treatment**	
Tumor resection	50
Chemotherapy	14
**Histologic subtype**	
Well/Moderate	12
Poor	39
Signet ring cell carcinomas	9
Undetermined	4
**Location**	
Proximal	32
Distal	32
**SUVmax**	
Mean ± SD	8.0±5.3
Range	1.8-27.7
**PD-L1 expression**	
Negative	57
Positive	7
**PD-L1-TILs expression**	
Negative	39
Positive	25

### Correlation between SUVmax and PD-L1/PD-L1-TILs expression

**Figure 1:**
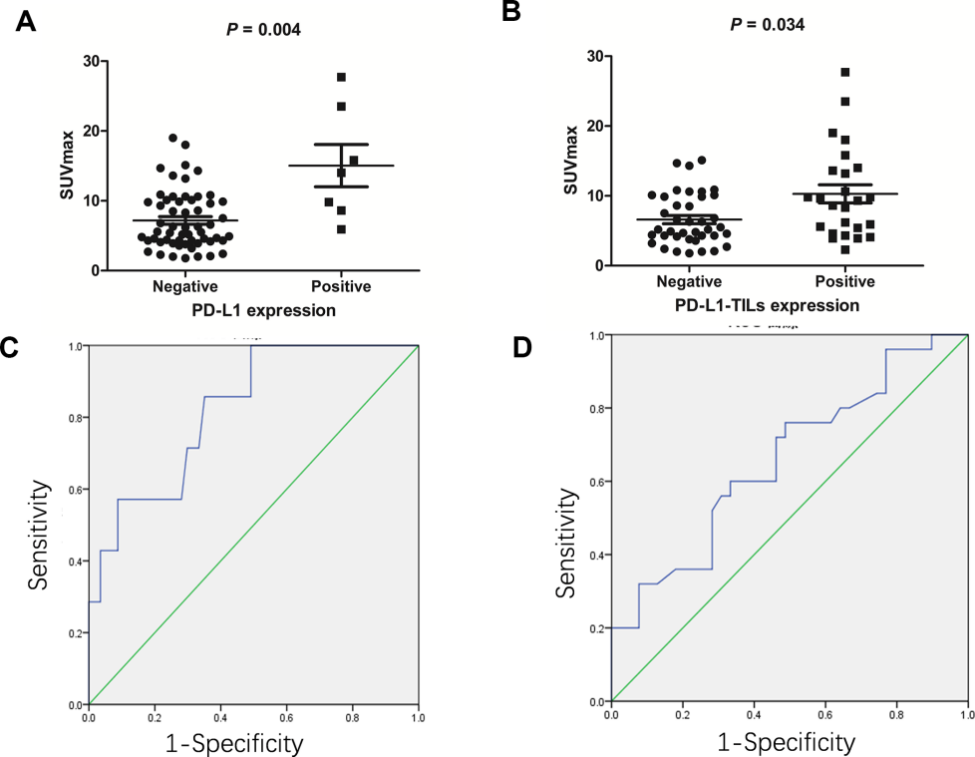
**The association between ^18^F-FDG accumulation and PD-L1/PD-L1-TILs status in gastric cancer (n=64).** (**A**) The association between ^18^F-FDG accumulation and PD-L1 status. Gastric cancers with positive PD-L1 had higher SUVmax compared with those lacking PD-L1 (15.0 ± 8.0 vs. 7.2 ± 4.2, respectively; P = 0.004). (**B**) The association between ^18^F-FDG accumulation and PD-L1-TILs status. Gastric cancers with positive PD-L1-TILs had higher SUVmax compared with those lacking PD-L1-TILs (10.3 ± 6.5 vs. 6.6 ± 3.7, respectively; P = 0.034). (**C**) ROC analysis of SUVmax for predicting PD-L1 status. When the cutoff threshold of SUVmax was 8.55, the sensitivity and specificity to predict PD-L1 status was 85.7% and 64.9%, respectively. The area under curve was 0.822 (95% CI: 0.674-0.97; P = 0.006). (**D**) ROC analysis of SUVmax for predicting PD-L1-TILs status. When the cutoff threshold of SUVmax was 7.9, the sensitivity and specificity to predict PD-L1-TILs was 60.0% and 66.7%, respectively. The area under curve was 0.658 (95% CI: 0.52-0.796; P = 0.034).

We investigated PD-L1/PD-L1-TILs status by immunohistochemical analysis (n=64). In the primary tumors we identified a positive association between SUVmax and the status of PD-L1 (Figure 1A) and PD-L1-TILs (Figure 1B). Tumors with positive expression of PD-L1 had higher SUVmax compared with those lacking PD-L1 expression (15.0 ± 8.0 vs. 7.2 ± 4.2, respectively; P = 0.004). Tumors with positive expression of PD-L1-TILs also had higher SUVmax compared with those lacking PD-L1-TILs expression (10.3 ± 6.5 vs. 6.6 ± 3.7, respectively; P = 0.034).

We next determined the SUVmax threshold to predict the status of PD-L1/PD-L1-TILs. ROC analysis demonstrated that the highest accuracy (67.2%) to predict PD-L1 status was obtained when the SUVmax threshold was 8.55, resulting in area under curve of 0.822±0.075. The sensitivity and specificity for the prediction of PD-L1 status was 85.7% (6/7) and 64.9% (37/57), respectively (Figure 1C). Likewise, ROC analysis also demonstrated that the highest accuracy (64.2%) for the prediction of PD-L1-TILs expression was obtained with a SUVmax cutoff value of 7.9, resulting in area under curve of 0.658± 0.07. Similarly, the sensitivity and specificity for the prediction of PD-L1-TILs status was found to be 60.0% (15/25) and 66.7% (26/39), respectively (Figure 1D).

### Correlation between clinicopathologic characteristics and PD-L1/PD-L1-TILs status

Patients were separated into two groups on the basis of PD-L1/PD-L1-TILs status. The relationship between clinicopathologic characteristics in patients treated with gastrectomy and PD-L1/PD-L1-TILs expression were evaluated (n=50) (Table 2). No significant differences in gender, tumor location and size, vascular invasion, T stage, N stage, distant metastasis, histologic type were observed between PD-L1-positive and PD-L1-negative groups. Whereas, the SUVmax of the primary tumors was significantly different between these two groups. Similar correlations were also observed for PD-L1-TILs expression (Table 2).

**Table 2:** Relationship between PD-L1/PD-L1-TILs expression and clinicopathological characteristics in gastric cancer (n=50).

**Variable**	**Total**	**PD-L1 expression**		**χ2**	**P value**	**PD-L1-TIL expression**		**χ2**	**P value**
		**Negative**	**Positive**			**Negative**	**Positive**		
**Gender**									
Male	35	32	3	0.265	0.629	9	6	0.001	1
Female	15	13	2			21	14		
**Age (years)**		60.8±14.3	65.4±7.9		0.484	60.3±12.6	62.7±12.6	0.565
**Tumor size (cm)**		5.2±2.8	5.7±2.9		0.738	5.2±2.7	5.5±3.0		0.738
T category									
T1/T2	16	15	1	0.368	0.544	9	7	0.138	0.71
T3/T4	34	30	4			21	13		
**N stage**									
0	11	10	1	3.941	0.628	5	6	7.07	0.07
1	13	10	3			6	7		
2	12	12	0			11	1		
3	14	13	1			8	6		
**Distant metastasis**									
No	37	35	2	3.338	0.103	23	14	0.277	0.599
Yes	13	10	3			7	6		
**Histologic type**									
Well/Moderate	12	12	0	1.759	0.415	9	3	1.701	0.427
Poor	30	26	4			16	14		
Signet ring cell carcinomas	8	7	1			5	3		
**Vascular invasion**									
No	14	12	2	0.397	0.611	7	7	0.81	0.368
Yes	36	33	3			23	13		
**Location**									
Proximal	27	25	2	0.438	0.651	16	11	0.013	0.908
Distal	23	20	3			14	9		
**SUVmax**		7.0±4.1	12.7±7.0		0.046	6.2±3.3	9.7±5.6		0.038

In the multivariate analysis including factors with a *P* value of 0.2 or less, only the SUVmax of primary tumors remained significantly associated with PD-L1 status [Table 3; OR, 1.2; 95% CI, 1.01–1.33; P = 0.043]. Similarly, in the multivariate analysis including factors with a *P* value of 0.2 or less, only the SUVmax of primary tumors remained significantly associated with PD-L1-TILs status [Table 3; OR, 1.3; 95% CI, 1.05–1.5; P = 0.016]. Taken together, these above results demonstrate that SUVmax may be used to predict PD-L1/PD-L1-TILs status in gastric cancer.

**Table 3:** Multivariate analysis of PD-L1 and PD-L1-TILs expression in patients with gastric cancer (n=50).

**Predictors**	**Factor**	**Odds ratio**	**OR (95% CI)**	**P**
PD-L1	SUVmax	1.2	1.01-1.53	0.043
	Distant metastasis	5.7	0.6-49.7	0.116
PD-L1-TIL	SUVmax	1.3	1.05-1.5	0.016
	N stage	0.58	0.3-1.8	0.086

## DISCUSSION

Immune checkpoint blocker has been widely used for treatment of metastatic or recurrent advanced gastric cancer [7]. The status of PD-L1 is being explored as a predictive marker for response to anti-PD-L1 blocker [8, 16]. Detecting PD-L1 expression is now common in the management of gastric cancer. ^18^F-FDG PET/CT is a noninvasive diagnostic tool to detect malignant tumors. Though several studies have suggested the association between SUVmax and PD-L1 status in lung cancer [17, 18], and our previous study have demonstrated the association between SUVmax and the status of PD-L1 in bladder cancer [19], but possible underlying mechanisms are still unclear. In the current study we demonstrate that gastric cancers with positive expression of PD-L1/PD-L1-TILs had higher SUVmax compared with those lacking PD-L1/PD-L1-TILs expression. To our knowledge, this is the first study that analyzes the correlation between ^18^F-FDG accumulation and PD-L1/PD-L1-TILs status in gastric cancer patients.

Immunotherapy was widely used for treating malignant tumors [7, 20, 21]. However, the clinicopathologic characteristics of patients correlated with response to immune checkpoint blocker are still unknown, and selecting the patients who are possible to achieve response from targeting PD-L1 and excluding those who are unresponsive to the immunotherapy is still an important question. The status of PD-L1/PD-L1-TILs was often assessed by immunohistochemistry analysis [22]. Whereas, tumor tissue obtained by gastroscopy or surgical resection are invasive. For these reasons, other noninvasive methods, such as ^18^F-FDG PET/CT, which could predict the expression of PD-L1/PD-L1-TILs and inform optimal treatment decision with anti-PD-L1 antibodies would be of important clinical value in gastric cancer patients.

In our study we discovered a positive association between SUVmax and PD-L1/PD-L1-TILs status in gastric cancers. The ROC curves analysis demonstrated that ^18^F-FDG accumulation of primary tumors could be useful for predicting PD-L1/PD-L1-TILs status. Multivariate analysis revealed that SUVmax was the only significant predictor of PD-L1/PD-L1-TILs status in gastric cancers. However, the molecular mechanism of association between ^18^F-FDG accumulation and PD-L1/PD-L1-TILs status is still unclear. HIF1α played a key role in regulating ^18^F-FDG accumulation of tumor cells [15, 23]. In addition, HIF-1α was a transcription factor of PD-L1 and could upregulate PD-L1 expression [24]. These studies demonstrated that the positive association between ^18^F-FDG accumulation and PD-L1/PD-L1-TILs expression may be a reflex of the HIF-1α activation. Pearce EL et.al [25] show that PD-L1 blockade by the PD-L1 antibody could significantly inhibit the AKT pathway, leading to the suppressed translation of glycolytic related enzymes, demonstrating that PD-L1 was the regulation factor of ^18^F-FDG accumulation in tumor cells. In addition, previous studies show that peroxisome proliferator-activated receptor-gamma (PPAR-gamma) has been implicated in regulating ^18^F-FDG [26] and the PD-L1 expression [27]. So the association between ^18^F-FDG and the PD-L1 expression may also be a reflex of the PPAR-gamma pathway activation.

Novel immunotherapeutic methods are being advanced to suppress the expression of PD-L1. For these reasons, noninvasive strategies, including molecular imaging tools, which could be used for predicting the status of PD-L1/PD-L1-TILs, are of important clinical value, and have good prediction effect on the response to anti-PD-L1 blocker.

This study is limited by its small sample and retrospective design. Though ^18^F-FDG PET/CT could have a good predictive value, it is not feasible to obtain an optimal cutoff for SUVmax in the clinical setting, and ^18^F-FDG PET/CT cannot supersede immunohistochemistry analysis for detecting PD-L1/PD-L1-TILs expression. In addition, because of the frequency of physiological ^18^F-FDG uptake and inflammation induced ^18^F-FDG uptake, sometimes it is hard to identify the ^18^F-FDG uptake from the gastric tumor itself. And there is a partial overlap between positive and negative PD-L1/PD-L1-TILs expression cases in ^18^F-FDG uptake. However, this study can promote the advancement of noninvasive methods to infer PD-L1/PD-L1-TILs status. Progress in new radiotracers may improve the accuracy of this technique.

## CONCLUSIONS

Gastric cancer with positive PD-L1/PD-L1-TILs expression have elevated ^18^F-FDG accumulation. ^18^F-FDG PET/CT has the ability to become a useful method to assess the molecular phenotypic information of gastric cancer, and have good prediction effect on the response to anti-PD-L1 blocker in gastric cancers. Additional prospective and large studies are required to verify our results and evaluate if molecular imaging can be useful for predicting the status of PD-L1 in gastric cancers, as well as for assisting the treatment decision making on when to employ anti-PD-L1 blocker therapies.

## PATIENTS AND METHODS

### Population

64 patients with gastric cancer (20 women and 44 men; age: 26–84 y) were involved in our study. The participants underwent ^18^F-FDG PET/CT imaging before surgical resection (n=50) or chemotherapy (n=14) at the RenJi Hospital between December 2016 and May 2019. Inclusion criteria were as follows: treatment with radical gastrectomy or chemotherapy; immune therapy had not been administered before scan; all patients was confirmed by pathology of gastroscopy or surgical tumors; clinicopathological data were all available, including tumor location, tumor size, vascular invasion, N stage, T category, histologic subtype; and tissue specimens were available for immunohistochemical staining. Informed consent was not obtained, and the RenJi Hospital Institutional Review Board approved this retrospective study.

### ^18^F-FDG PET/CT scan

Gastric cancer patients had been asked to fast for more than six hours before ^18^F-FDG was injected. Patients’ glucose levels were measured before ^18^F-FDG administration, and in this study there were no patients whose blood glucose level exceeded 140 mg/dL. The mean accumulation time was approximately 60 minutes. PET was carried out with a combined PET/CT. The CT was used for attenuation correction.

Two board-certified nuclear medicine physicians assessed the ^18^F-FDG accumulation. ROIs were placed on the tumor uptake lesion of PET imaging for semi-quantitative analysis. The following formula was used to calculating SUVmax of the primary tumor: decay-corrected tracer tissue concentration /(injected ^18^F-FDG dose /patients’ weight).

### Immunohistochemical analysis

Tumor tissues were paraffin-embedded and used for immunohistochemical analysis. The markers CK (cytokeratin) and LCA (the lymphocyte common antigen) were used to differentiate tumor cells and tumor infiltrating lymphocytes. Positivity for PD-L1 (indicating tumor cells) or PD-L1-TILs (indicating tumor infiltrating lymphocytes) was assessed by one board-certified pathologist. The positive percentage of stained cells covered by PD-L1 and PD-L1-TILs was quantified. Cases with >1% positive percentage of PD-L1/PD-L1-TILs were considered as high expression.

### Statistical analysis

All values are demonstrated as mean ± SD. The statistical differences between different groups were compared using Mann–Whitney U test or chi-square test. SPSS software was used for statistical analysis.
